# Is lung ultrasound score a useful tool to monitoring and handling moderate and severe COVID-19 patients in the general ward? An observational pilot study

**DOI:** 10.1007/s10877-021-00709-w

**Published:** 2021-05-04

**Authors:** Marco Baciarello, Andrea Bonetti, Luigi Vetrugno, Francesco Saturno, Antonio Nouvenne, Valentina Bellini, Tiziana Meschi, Elena Bignami

**Affiliations:** 1 Anesthesiology, Department of Medicine and Surgery, Critical Care and Pain Medicine Division, University of Parma, Viale Gramsci 14, 43126 Parma, Italy; 2 Anesthesiology and Critical Care Division, Azienda Ospedaliero-Universitaria Di Parma, Parma, Italy; 3 Department of Medicine, Anesthesia and Intensive Care Clinic, University of Udine, Udine, Italy; 4 Geriatric-Rehabilitation Department, Azienda Ospedaliero-Universitaria Di Parma, Parma, Italy; 5 Department of Medicine and Surgey, University of Parma, Parma, Italy

**Keywords:** Lung Ultrasound, COVID-19, Computed tomography, Non invasive ventilation

## Abstract

Lung ultrasound is a well-established diagnostic tool in acute respiratory failure, and it has been shown to be particularly suited for the management of COVID-19-associated respiratory failure. We present exploratory analyses on the diagnostic and prognostic performance of lung ultrasound score (LUS) in general ward patients with moderate-to-severe COVID-19 pneumonia receiving O_2_ supplementation and/or noninvasive ventilation. From March 10 through May 1, 2020, 103 lung ultrasound exams were performed by our Forward Intensive Care Team (FICT) on 26 patients (18 males and 8 females), aged 62 (54 – 76) and with a Body Mass Index (BMI) of 30.9 (28.7 – 31.5), a median 6 (5 – 9) days after admission to the COVID-19 medical unit of the University Hospital of Parma, Italy. All patients underwent chest computed tomography (CT) the day of admission. The initial LUS was 16 (11 – 21), which did not significantly correlate with initial CT scans, probably due to rapid progression of the disease and time between CT scan on admission and first FICT evaluation; conversely, LUS was significantly correlated with PaO_2_/FiO_2_ ratio throughout patient follow-up [R = − 4.82 (− 6.84 to − 2.80; p < 0.001)]. The area under the receiving operating characteristics curve of LUS for the diagnosis of moderate-severe disease (PaO_2_/FiO_2_ ratio ≤ 200 mmHg) was 0.73, with an optimal cutoff value of 11 (positive predictive value: 0.98; negative predictive value: 0.29). Patients who eventually needed invasive ventilation and/or died during admission had significantly higher LUS throughout their stay.

## Introduction

Lung ultrasound (LU) imaging has a crucial role in the management of COVID-19-associated pneumonia [1]. Although chest tomography (CT) is the reference standard for diagnosis, LU has been shown to be useful in the pre-hospital setting [2] and upon hospital admission of COVID-19 patients [3, 4]. Respiratory COVID-19 symptoms may persist for at least 60 days in 67% of patients [5], and while repeat CT may be impractical and/or unsafe for patients and operators, LU may be the default imaging modality for monitoring patients’ condition throughout their hospital stay and, if needed, after discharge [6]. Agreement with CT seems to be more than adequate in SARS-CoV-2-related pneumonia [7, 8].

Lung ultrasound score (LUS) is a semi-quantitative model which entails the assessment of 12 regions for the presence of specific artifacts caused by increased extravascular water and/or loss of aeration. The score ranges from 0 (healthy lung) through a theoretical 36 (consolidations in all regions) [9].

During the first surge in COVID-19 cases in Northern Italy, between February and April 2020, medical wards and intensive care units were overwhelmed by the sheer number of respiratory failure patients requiring admission and supportive treatment [10]. Among the rearrangement of hospital activities to meet the increased demand, we instituted a forward intensive care team (FICT), a team of intensivists providing regular consultations and management of patients with moderate to severe COVID-19 pneumonia. The FICT was meant to support patients who were at risk for admission to Intensive Care Unit (ICU) for mechanical ventilation, by administering high-flow nasal cannula (HFNC) O_2_ and noninvasive ventilation on medical floors. The goal was to provide patients who were candidates for ICU admission with effective treatments, so that they might better endure the wait for bed availability or avoid ICU admission altogether in case of sustained improvement.

In this study, we present the results of our analysis of consecutive cases followed by our FICT, who were monitored with repeat LUS examination as well as more conventional parameters. The aim of the study is to explore the diagnostic and prognostic usefulness of LUS in terms of risk of admission to the ICU and/or in-hospital death.

## Material and methods

This was an observational retrospective pilot study aimed at comparing lung ultrasound examination, computer tomography scan and gas exchange when all exam where available. The study was approved by the Local Ethics Committee (protocol nr. 730/2020, Comitato Etico Unico per l’Area Vasta Emilia Nord). Patients were routinely asked to consent to the use of anonymized aggregate data for research purposes as part of the intake process, as customary at our institution. We checked for expressed consent in patients’ medical records; additionally, we attempted to contact survivors to discharge to confirm their consent to the use of clinical data for the present study.

As a convenience sample, we reviewed medical records of the first 30 patients followed by the FICT between March and April 2020. Indications for hospital admission included persistently high fever, SpO_2_ ≤ 92% and/or < 90% after a walk test, in the presence of signs and symptoms compatible with viral pneumonia. All patients in COVID-19 medical wards at our hospital had at least two positive polymerase chain-reaction tests for SARS-CoV-2. Patients had been referred to the FICT referral by their treating physicians due to respiratory compromise, and specifically for failure to reach and maintain a peripheral blood oxygen saturation (SpO_2_) ≥ 93% and/or a respiratory rate (RR) ≤ 30 breaths per minute despite optimal therapy and O_2_ ≤ 15 L/min through a non-rebreather mask. Referrals were followed on a regular basis by FICT physicians until clinical improvement (i.e., return to O_2_ supplementation < 15 L/min) or terminal deterioration (withdrawal of care), or until transfer to the ICU if applicable. Consultations could result in initiation of HFNC therapy, continuous positive airway pressure (CPAP) or noninvasive ventilation (NIV). Patients were defined as being co-managed by the FICT between the first and last assessment. In each case the decision to switch patients to NIV was based on clinical criteria including respiratory rate greater than 30 breaths per minute, dyspnea, peripheral oxygen saturation less than 91% and PaO_2_/FiO_2_ ratio less than 200 mmHg.

### Lung ultrasound

Lung ultrasound examinations were performed whenever feasible in patients followed by the FICT, typically no less than once every other day. Both took almost 50% of total exams each. The scanning technique has been described before [11]; briefly, each hemithorax was divided into three regions: anterior (ventral to the anterior axillary line), middle (between the anterior and posterior axillary lines) and posterior (dorsal to the posterior axillary line); each region was further divided into an upper and lower zone by a transverse plane passing through the xyphoid process. The worst scan from each zone was scored as follows: A-lines or < 2 separate B lines (normal or A-pattern, plus lung sliding), 0 points; well-spaced B lines (B-pattern, plus lung sliding), ≥ 3, 1 point; coalescent or fused B-lines (light beam, plus lung sliding), 2 points; lung consolidation, including multiple small subpleural consolidations, 3 points. All examinations were performed with the same device (iQ ultrasound probe, Butterfly Network, Inc., Guilford, CT, USA) and stored using the original software in a dedicated, secured cloud store system.

All FICT practitioners underwent a lung ultrasound course upon starts the resident program. Data from ultrasound examinations was recovered from the ultrasound device’s database and analyzed by two authors (A.B. and F.S.); in case of discordance, a third Author (M.B.) was asked to break the tie by convening with either assessment. All exams were performed by one of three physicians who had performed at least 25 lung ultrasound examinations [12].

On admission, all the patients routinely underwent non-contrast chest high-resolution computed tomography (HRCT), performed with either a 128-slice scanner (SOMATOM Definition Edge, Siemens Healthineers, Erlangen, Germany) or (during peak periods) an extra 16-slice truck-mounted mobile scanner (SOMATOM Emotion, Siemens Healthineers, Erlangen, Germany). Images were acquired with the patient in the supine position during end-inspiration breath-hold. Extent of lung involvement, as typical ground glass areas, was scored as percentage of the whole lungs, by 5% of discrete increment (range 0 to 100), while consolidations, another typical finding in SARS-CoV-2 pneumonia, were described with the aim of differentiating CT pattern compatible with organizing phenomena as opposed to other non-specific consolidation patterns.

Routine arterial blood gas (ABG) analyses were drawn in the morning, with patients maintaining on the prescribed oxygen supplementation/mechanical ventilation mode. We defined moderate-severe COVID-19 pneumonia as one leading to PaO_2_/FiO_2_ ratio ≤ 200 mmHg; severe disease was defined by PaO_2_/FiO_2_ ≤ 100 mmHg.

### Statistical analysis

Data were analyzed using non-parametric and/or robust approaches and presented as median (interquartile range) or count (percentage); where applicable, 95% confidence intervals were computed using bias-corrected bootstrap approaches. A receiver operating characteristics curve was plotted for LUS as a marker of PaO_2_/FiO_2_ ratio ≤ 200 mmHg (“moderate-severe” disease). The threshold value for LUS was selected with Youden’s J statistic. We utilized both fixed-effects and mixed (random) effects models for linear (LUS vs. CT score) and logistic regression (LUS vs. risk of composite outcome). Regression terms were added in a stepwise fashion except for the fixed effect of LUS and a random between-subject effect (as intercept), which were kept constant; a random interaction term was also tested (subject × LUS). Regression terms were included in models if they led to a decrease in Akaike’s Information Criterion ≥ 1 unit, suggesting a ≥ 65% chance of the new model reducing information loss. Analyses were run using the *lme4* [13], *ggeffects* [14], *pROC* [15] and *plotROC* [16] packages for the R programming language [17]. A *p*-value < 0.05 was considered statistically significant.

## Results

From March 10 through May 1, 2020, 103 lung ultrasound exams were performed on 26 patients (18 males and 8 females), aged 62 (54 – 76) and with a BMI of 30.9 (28.7 – 31.5). The median patient follow-up was 8 (6 – 9) days, with a maximum duration of 31.9 days. Table 1 describes the characteristics of included patients. Seven patients (27%) were transferred to the ICU by the FICT, where all were tracheally intubated and ventilated. Of these patients, 4 died (57%). The overall study population mortality was 11 (42%).

**Table 1:** Characteristics of included patients

Age (y)	62 (54, 76)
Sex	
F	8 (31%)
M	18 (69%)
Body mass index (kg·m^-2^)	30.9 (28.7, 31.5)
Chest CT score (%)	42 (26, 68)
Length of hospital stay	26 (17, 35)
Deceased during stay	11 (42%)
Diabetes mellitus	5 (19%)
Chronic hypertension	13 (50%)
Chronic respiratory disease(s)	
0	25 (96%)
2	1 (3.8%)
Other chronic cardiovascular disease(s)	
0	24 (92%)
1	1 (4%)
2	1 (4%)
Other chronic metabolic disease(s)	2 (7.7%)

*1* Statistics presented: Median (IQR); n (%)

**Fig. 1:**
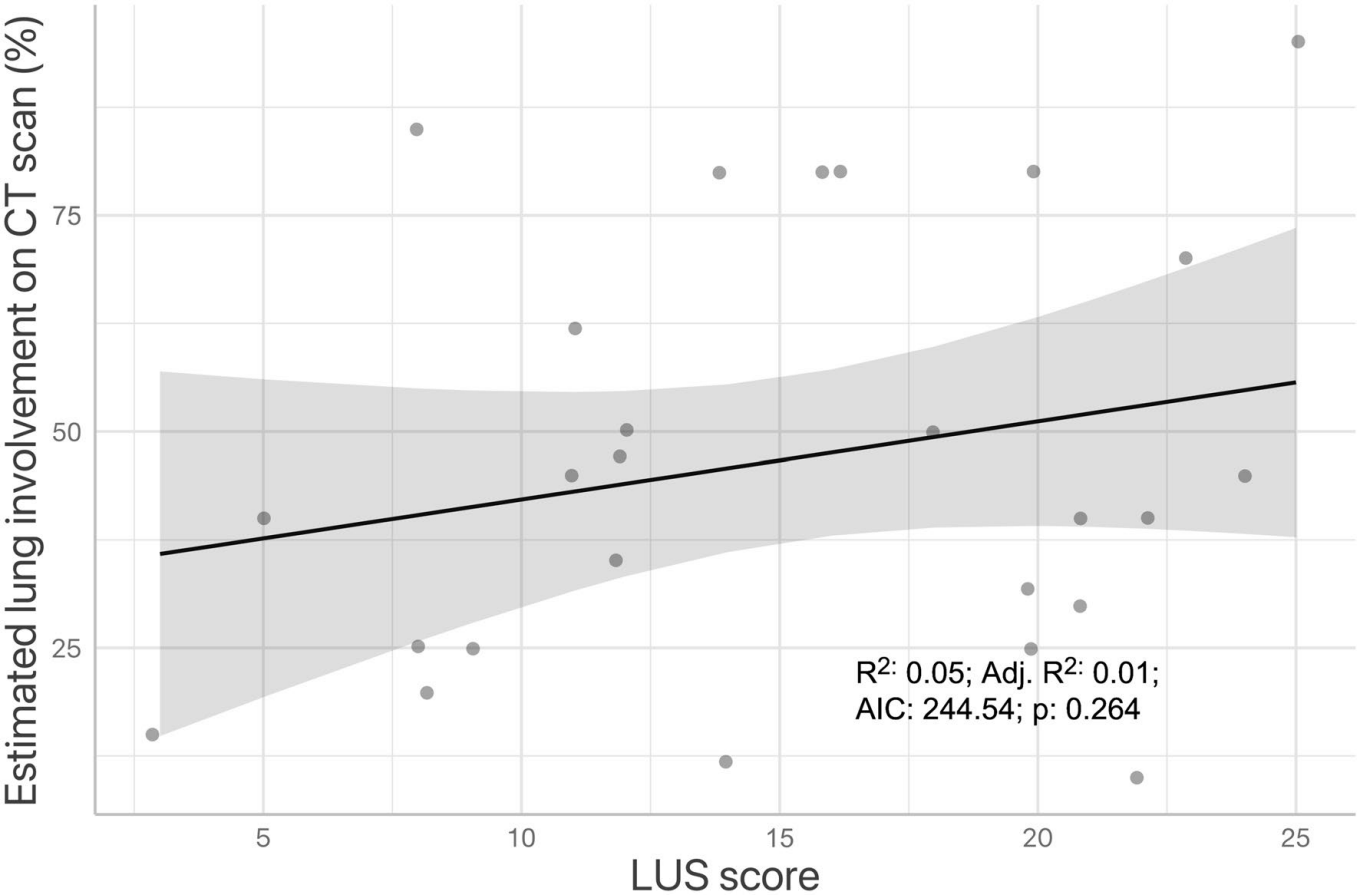
Linear correlation of lung ultrasound scores with the estimated proportion of lung volume involved with COVID-19 associated interstitial pneumonia; the first CT scan and chest ultrasonography results are considered. *CT* computed tomography

The first LU was performed by FICT on patients’ first evaluation, a median 6 (5 – 9) days after initial hospital admission; the initial LUS was 16 (11 – 21), and the values did not significantly correlate with admission CT scan findings (Fig. 1).

**Fig. 2:**
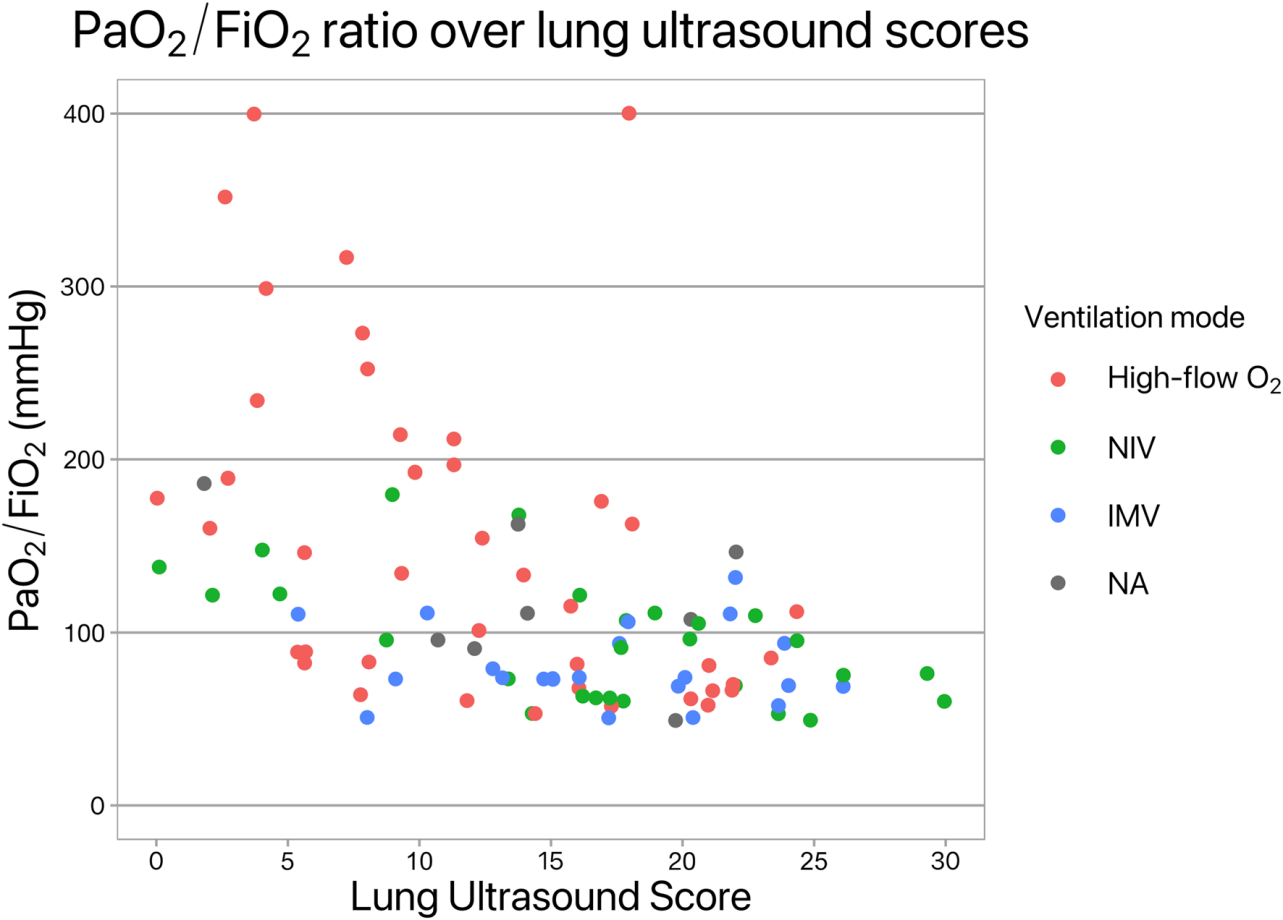
Scatterplot of lung ultrasound scores against PaO2/FiO2 ratio on arterial blood gas analyses, different colors indicate types of ventilatory support. *NIV* noninvasive ventilation, *IMV* invasive ventilation via orotracheal or tracheostomy tube, *NA* information not available for the data point

The initial ventilatory assistance mode upon FICT referral was HFNC O_2_ supplementation in 22 patients; NIV was immediately initiated in the other four patients. Seven (27%) patients were eventually admitted to an ICU and received invasive mechanical ventilation (IMV). Three patients who improved to PaO_2_/FiO_2_ ratio > 300 mmHg immediately after ventilatory support were rapidly weaned. Figure 2 shows the relationship between LUS and PaO_2_/FiO_2_ throughout patients’ stays classified by type of ventilatory assistance.

**Fig. 3:**
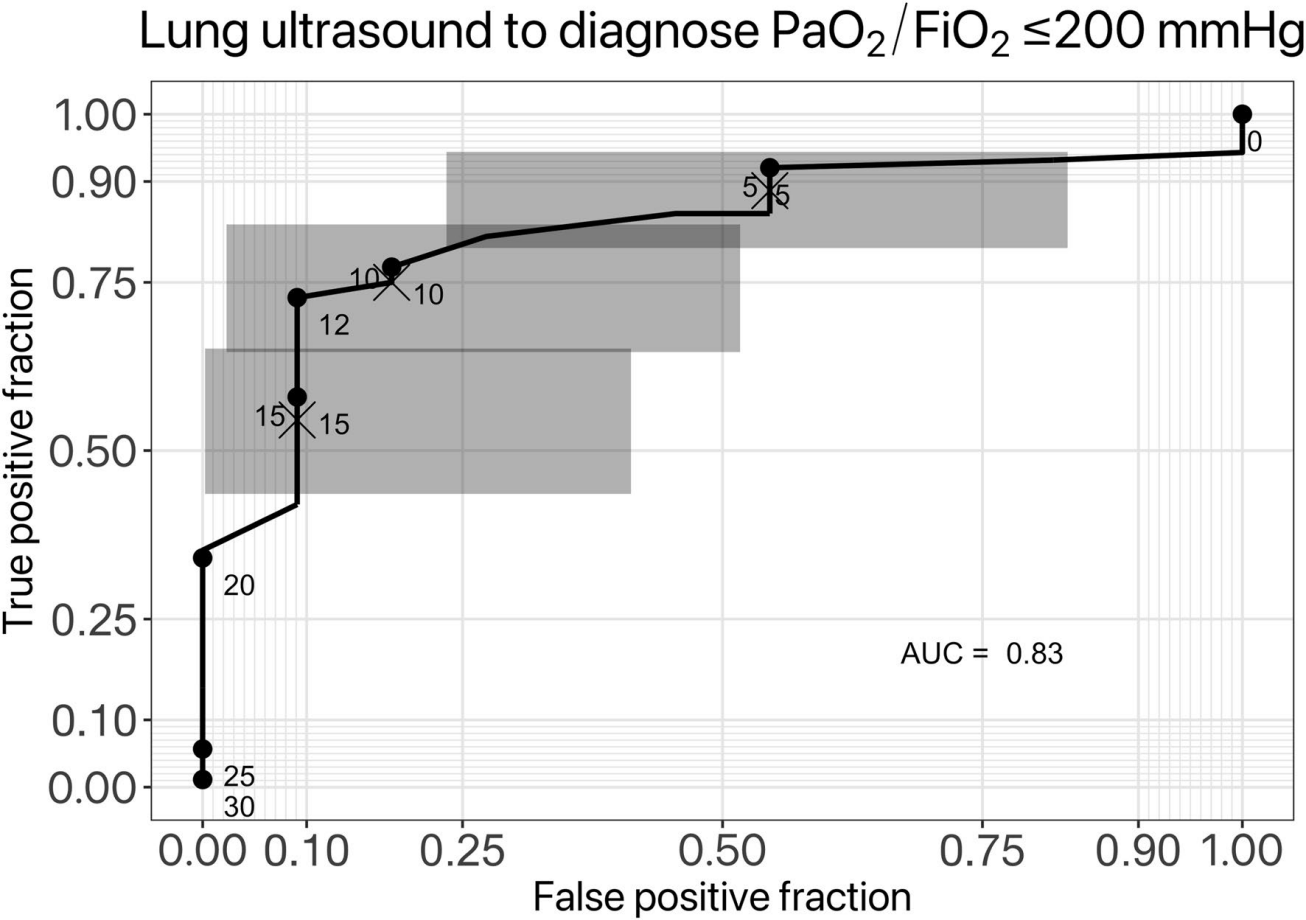
Receiver operating characteristics curve for the diagnosis of PaO2/FiO2 ≤ 200 mmHg with ultrasound score: points along the curve indicate arbitrary proposed cut-offs; shaded areas represent 95% confidence intervals for the curve in those segments

A LUS score of 11 was found to have a sensitivity of 0.73 (specificity 0.95; positive predictive value: 0.98; negative predictive value: 0.29) for the diagnosis of moderate-severe disease (PaO_2_/FiO_2_ ratio ≤ 200 mmHg); the full receiver operating characteristics curve for LUS with respect to moderate-severe COVID-19 respiratory failure is presented in Fig. 3. Values of LUS correlated with PaO_2_/FiO_2_ ratio throughout patient follow-up. We examined the correlation between LUS and the composite endpoint with both generalized and mixed effects logistic regression. In both cases, there was a significant correlation with PaO_2_/FiO_2_ ratios throughout the patients’ hospital stay. The most parsimonious and informative model according to AIC was one with fixed effects for LUS score and age, accounting for random inter-subject intercepts and LUS coefficients (Table 2); the introduction of time-related terms (admission day or admission week) did not improve the model fit significantly. According to this model the PaO_2_/FiO_2_ ratio will decrease approximately 3.66 mmHg for each additional LUS score point, and 1.4 mmHg for each additional year of age above the population mean at admission (Table 2). The interaction with time did not significantly improve the model (AIC: 1060, BIC: 1081). In the logistic regression model, LUS was the only significant risk factor for the composite outcome of ICU admission and/or in-hospital death; the mixed effects model, accounting for interpatient variability, was significantly more informative (20.5 vs. 28.0) but almost all of the variance was attributable to random between-subjects’ effects, which effectively voids it significance.

**Table 2:** Optimization of regression models for lung ultrasound score

Linear model				Mixed-effects mode		
Predictors	Estimates	CI	p	Estimates	CI	p
(Intercept)	285.18	222.06 to 348.30	** < 0.001**	264.97	179.41 to 350.53	** < 0.001**
LUSs	− 4.82	− 6.84 to − 2.80	** < 0.001**	− 3.66	− 6.15 to − 1.17	**0.004**
Age	− 1.43	− 2.43 to − 0.43	**0.006**	− 1.39	− 2.61 to − 0.17	**0.025**
Random effects						
σ^2^					2457.29	
τ00					10,042.68 _Subject_	
τ11					13.62 _Subject.LUS_	
ρ01					− 1.00 _Subject_	
N					25 _Subject_	
Observations		95			95	
R^2^/R^2^ adjusted		0.316/0.301			0.375/NA	
AIC		1078.783			1051.101	

The optimal models were found to be those including age as a fixed term, but not the hospital admission day. A linear mixed effects model accounting for intersubject variation in intercept and value of the LUS score parameter estimate, as a random effect, was found to be superior in terms of R2 and AIC. *AIC* Akaike’s information criterion, *CI* 95% confidence intervals

**Fig. 4:**
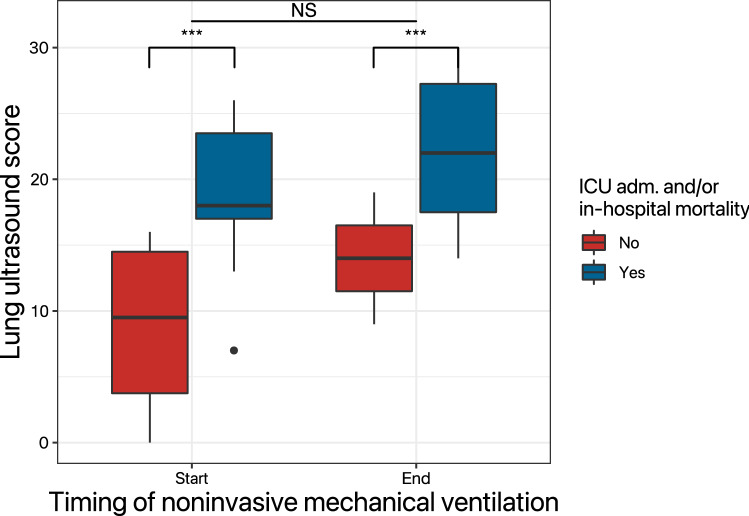
Lung ultrasound scores and oxygenation in patients undergoing NIV. Data are from the first (Start) and last (End) examination while receiving NIV. Patients are categorized according to outcome at the end of NIV treatment; the endpoint was defined as the combination of ICU admission for invasive ventilation and/or in-hospital death (whichever occurred first). Asterisks indicate statistically significant differences at p < 0.05. *ABG* arterial blood gas analysis, *ICU* intensive care unit, *NIV* noninvasive ventilation

Patients who went on to meet the composite outcome in our study (admission to an ICU bed and/or in-hospital death) had a LUS of 20 (16 – 23) when NIV was initiated, as compared to 12 (6 – 20) in patients who did not (p = 0.013). Figure 4 summarizes LUS scores upon their first and last examination during NIV (Table 3).

**Table 3:** Logistic regression models with and without random effects for the risk of ICU admission and/or in-hospital death

Fixed-effects model				Mixed-effects model		
Predictors	Odds ratios	CI	p	Odds ratios	CI	p
LUSs	1.28	1.08 – 1.65	0.016	5.64	1.19 – 26.68	0.029
Age	0.91	0.80 – 1.00	0.101	0.75	0.51 – 1.10	0.138
Random effects						
σ^2^					3.29	
τ00					4151.89 _Subject_	
ICC					1.00	
R2 Tjur		0.350			0.037/0.999	
AIC		28.026			20,534	

The most informative models according to AIC were those with LUSs and age as fixed effects terms; in the mixed effects model, addition of random between-subjects intercepts did improve the AIC but did not lead to improved model predictivity. The addition of other terms as specified in the Methods section did not significantly improve the AIC in either the fixed effects or mixed model. *AIC* Akaike’s information criterion, *ICU* intensive care unit

## Discussion

In March and early April 2020, Italy was the second country in the word after China to experience a surge in COVID-19 cases which overwhelmed several regional healthcare systems and led to oversaturation of ICU beds [10]. In this context, the University Hospital of Parma set up a FICT to help manage patients who were developing moderate to severe respiratory failure in medical wards and who could not immediately be admitted to ICUs, either because of borderline indications or due to temporary bed unavailability. In such a constrained-resource scenario, LUS examination has quickly became a standard feature of FICT evaluations [1], allowing to reduce the burden of disease monitoring on both patients (who could not be easily transferred to radiology suites), practitioners and the system as a whole.

The key results of this study are three: (i) in patients with COVID-19-associated pneumonia who were referred to a FICT for progressive worsening, LUS ≥ 12 points was associated with the prevalence of moderate-severe respiratory failure (AUC of the ROC curve: 0.83); (ii) a significant inverse relationship between LUS and PaO_2_/FiO_2_ ratio was found; (iii) patients who were admitted to the ICU for severe respiratory failure and/or who died during admission had higher LUS, and correspondingly lower PaO_2_/FiO_2_ ratio, irrespective of NIV use, as compared to those who did not require ICU admission and survived to discharge. Few studies have followed a population of COVID-19 patients with moderate-severe respiratory failure undergoing HFNC or NIV outside of the ICU.

Lung ultrasound may afford a semi-quantitative approach to ICU resource management, which can be integrated with other oxygenation parameters, or may substitute for data which are not readily available in all settings, such as arterial blood sampling and analysis. Our data show that LUS ≥ 12 is associated with moderate-severe disease; additionally, patients with negative outcome have significantly higher LUS both at the outset and end of NIV therapy outside of the ICU. Brahier et al. have recently published results of COVID-19 LUS screening the emergency department [18]. Their scoring system was different from classical LUS [19], and it was a median of 15 in patients who were eventually admitted to the ICU and/or died of respiratory failure; the AUC for the ROC when evaluating the risk of need for hospital admission in ED patients was found to be 0.77, similar to our 0.83.

The ability of LUS to anticipate clinical worsening has been proposed using a different scoring system in patients who were generally less severely affected than the ones in our study. In our population of sicker patients (mean PaO_2_/FiO_2_ ~ 180 mmHg, as opposed to 247 mmHg in Perrone et al.) those who went on to require invasive ventilation and/or die following NIV had significantly higher LUS than those who could remain on noninvasive assistance [20].

The absolute value of LUS in the negative outcome population of the present study [20 (16 – 23)] is quite similar to that found in studies utilizing the same scoring systems in different populations, despite the use of mechanical ventilation in the ward and in the ICU in the present study. For instance, the same scoring system was used in geriatric patients with no mention of ventilatory assistance [21] and a general population of COVID-19 patients, with or without overt respiratory failure [22]. In both cases, LUS ≥ 18 was associated with in-hospital mortality. In our opinion, this reinforces the general impression that ventilation does not modify the course of the disease.

Contrary to our previous findings and those of other authors [3, 8, 23, 24], LUS did not correlate significantly with CT score in this study population. This might be due to the different timing of CT and LU: these were performed within 24 h of admission in the cited studies, whereas in the present population LU was performed by intensivists on referral to the FICT, which happened 7 (5 – 10) days after admission. In those patients in the present series LUS were performed upon their first evaluation by the FICT, which happened 7 (5 – 10) days after admission. Moreover, we postulate that the relatively low sensitivity of our threshold for detection of moderate-severe disease using LUS may be due to the presence of a second pathological mechanism leading to hypoxia: microvascular thrombosis, which has been described extensively in *post-mortem* studies [25, 26], and does not necessarily lead to increased extravascular lung water and, thus, is not quantifiable on LU.

Our study is primarily limited by the relatively small, retrospective cohort of patients. Retrospective data in this context may be affected by selection bias, although these patients are representative of the population of assisted by our FICT. In a small population, individual effects are more evident. This is reflected in our regression models including random effects (*i.e.*, considering interindividual variations as an unpredictable factor), which show wide confidence intervals and high intraclass correlation coefficients. A larger, prospective cohort would be advisable for more significant results. Another limitation is that we did not account for interobserver variability in LU scoring, although we assume that a clear-cut scoring system and adequate operator experience may have minimized it [27].

In summary, we present results of our analyses on the clinical usefulness of LUS during a major COVID-19 outbreak, in the setting of a surge in hospital admissions and ICU overload. In patients admitted to medical wards, LUS was useful in identifying and monitoring those with persistent PaO_2_/FiO_2_ ≤ 200 mmHg; LUS was significantly higher in patients who were eventually transferred to the ICU for intubation and IMV and/or who died. Prospective research will hopefully improve these results and determine appropriate LUS risk thresholds, in order to improve patient care and ICU utilization.
